# Transcriptomic profiling of near-isogenic lines reveals candidate genes for a significant locus conferring metribuzin resistance in wheat

**DOI:** 10.1186/s12870-023-04166-2

**Published:** 2023-05-05

**Authors:** Rudra Bhattarai, Hui Liu, Kadambot H.M. Siddique, Guijun Yan

**Affiliations:** 1 UWA School of Agriculture and Environment, The University of Western Australia, 6009 Perth, WA Australia; 2 The UWA Institute of Agriculture, The University of Western Australia, 6009 Perth, WA Australia

**Keywords:** Wheat, Near-isogenic lines, Metribuzin resistance, Differential gene expression, Marker-assisted selection

## Abstract

**Background:**

Weeds reduce wheat yields in dryland farming systems. Herbicides such as metribuzin are commonly used to control weeds. However, wheat has a narrow safety margin against metribuzin. Standing crops such as wheat with weeds in the same field can also be killed by the same dose of metribuzin. Therefore, it is important to identify metribuzin resistance genes and understand the resistance mechanism in wheat for sustainable crop production. A previous study identified a significant metribuzin resistance wheat QTL, *Qsns.uwa.4 A.2*, explaining 69% of the phenotypic variance for metribuzin resistance.

**Results:**

Two NIL pairs with the most contrasting performance in the metribuzin treatment and different in genetic backgrounds were compared using RNA sequence analysis, identifying nine candidate genes underlying *Qsns.uwa.4 A.2* responsible for metribuzin resistance. Quantitative RT-qPCR further validated the candidate genes, with *TraesCS4A03G1099000* (nitrate excretion transporter), *TraesCS4A03G1181300* (aspartyl protease), and *TraesCS4A03G0741300* (glycine-rich proteins) identified as key factors for metribuzin resistance.

**Conclusion:**

Identified markers and key candidate genes can be used for selecting metribuzin resistance in wheat.

**Supplementary Information:**

The online version contains supplementary material available at 10.1186/s12870-023-04166-2.

## Background

Wheat is a major cereal crop worldwide and one of Australia’s most significant agricultural trade commodities. However, weed infestation costs Australian agriculture AUD 2.5–4.5 billion per annum [1], equivalent to ~ 20% of the gross value. Weeds are a major problem in Mediterranean agriculture [2, 3] and generally grow actively during the wet winter season. Weed control is essential to ensure crops can fully use stored summer rainfall and prevent weed seeds from contaminating crop seeds. Controlling weeds using broad-spectrum herbicides such as metribuzin, is common in agriculture [4]. Metribuzin—a triazine herbicide that targets PS-II components—is used primarily as a pre-emergent herbicide [4]. In Western Australia, pre-emergent application of Metribuzin controlled 80% of germinating weeds in demonstration trials [5]. However, other factors such as resistant sources (varieties), soil type, herbicide dose, field moisture, ambient temperature, and growing degree days [6] can determine the effectiveness of herbicides. Despite having limitations to use herbicides, herbicides are regularly used in controlling weeds. While using herbicide such as metribuzin for controlling weeds it can also kill germinating wheat from the same field. Therefore, it is essential to investigate wheat’s metribuzin resistance traits and underlying genes.

RNA sequencing of NILs can identify differentially expressed genes in wheat, as isolines of each NIL pair are essentially fixed, thus differences between isolines from a locus or few loci can be obtained accurately by analysing the NILs. Comparing isolines of different genetic backgrounds ensures the identification of genes common to different NIL pairs; as both NIL pairs are contrasting for the same locus. At the same time, it helps reduce background noises. Therefore, NIL analysis using targeted QTL could be the best for characterising metribuzin resistance.

QTL for metribuzin resistance have been mapped on chromosome 4 A in two wheat populations: Chuan Mai 25 (Resistant, R) × Ritchie (Susceptible, S) [7] and Synthetic W7984 × Opata M85 [8] populations. The genetic distance of the QTL is 49.6–58.3 cM between two flanking markers, Xbarc170 and Xbarc350, with a physical distance of 130.84 Mb according to the wheat reference genome [9]. The original QTL region contains ten SSR markers, apart from the flanking markers [8]. However, QTL markers may not be suitable for direct gene characterisation, pyramiding and marker-assisted programs [10]; developing NILs using a heterogeneous inbred family method can ensure NIL pairs with different genetic backgrounds, as each pair is a descendent progeny from an F2 individual [11] such contrasting NIL pairs in comparative analysis can help to establish accurate marker-trait association. Several studies have used this method to develop NILs and characterise important major QTL through transcriptome profiling, thus refining the mapping resolution of the targeted QTL [12–14] for increasing selection accuracy. This study investigated differentially expressed genes (DEGs) and molecular markers for metribuzin resistance from two NIL pairs from different genetic backgrounds, allowing us to detect the most significant candidate genes and underlying marker-trait associations.

Our previous study identified an SSR marker (Xbarc343) from *Qsns.uwa.4 A.2* in a Synthetic W7984 ⋅ Opata M85 population, which was used to develop NILs in a Chuan Mai 25 ⋅ Ritchie population. Phenotypic characterisation confirmed that the resistance (R) allele from R-isolines increased metribuzin resistance by 63–85% (average 69%) compared with the susceptibility (S) allele from S-isolines. Thousand-grain weight co-segregated with metribuzin resistance, signifying the importance of this locus and its underlying gene(s) [15]. Similarly, NILs (NIL_3 and NIL_17) with different genetic backgrounds were used in RNA sequencing and real-time quantitative polymerase chain reaction (RT-qPCR) between isolines to identify key candidate genes and molecular markers for metribuzin resistance. This study aimed to (1) identify DEGs as candidate genes underlying *Qsns.uwa.4 A.2*, (2) explore the mechanism of metribuzin resistance by analysing the networks of the candidate genes, and (3) identify molecular variants and candidate genes for metribuzin resistance selection in wheat.

## Results

### Differentially expressed genes (DEGs) common between two NIL pairs

**Fig. 1:**
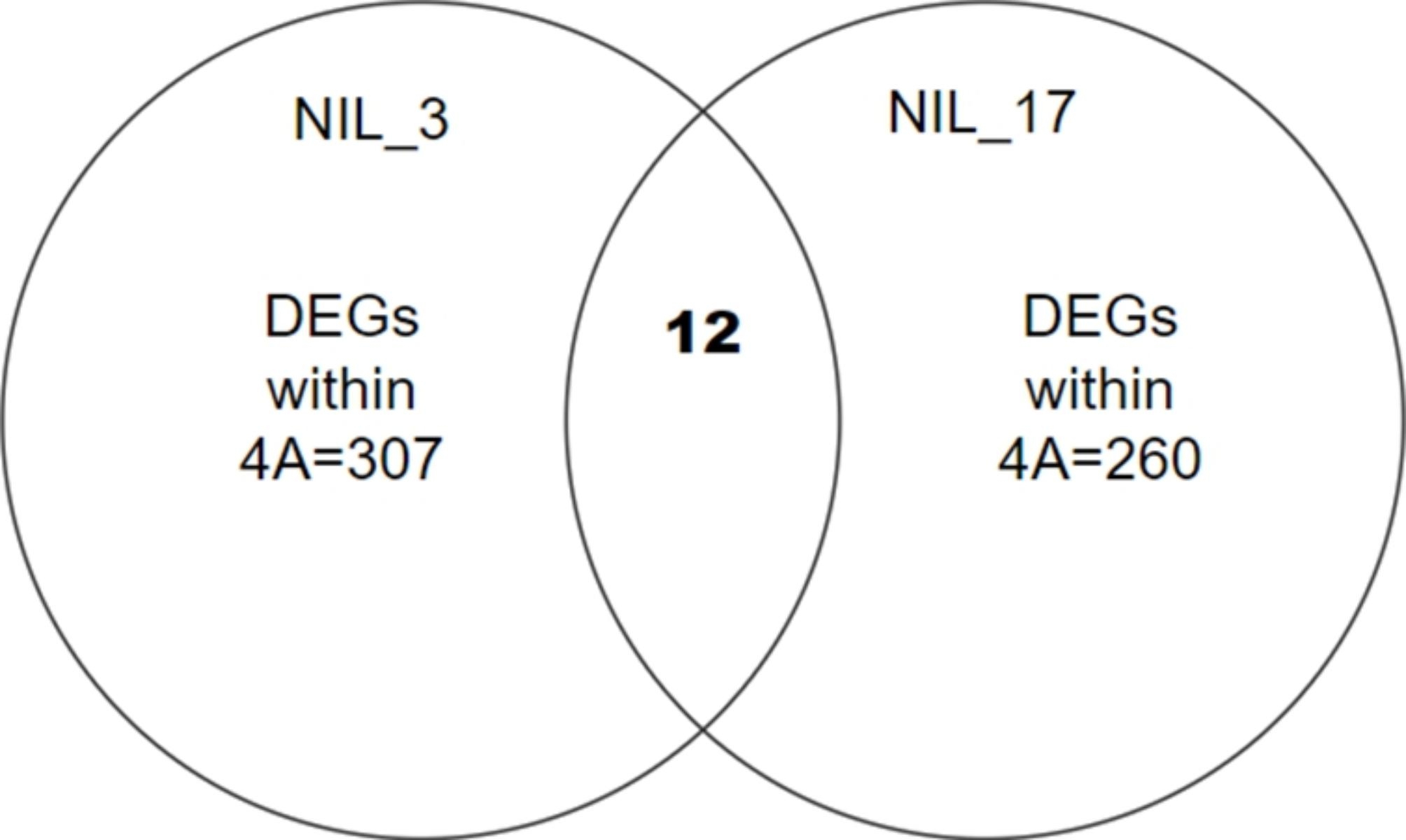
Twelve common DEGs were detected on chromosome 4 A between NIL_3 and NIL_17 as putative candidate genes responsible for metribuzin tolerance in wheat

Within the control group, 199 genes were detected across both NIL pairs, while 297 genes were detected across the NILs within the treatment group, including DEGs (significant expression at P≤0.05) and non-DEGs (differences in gene expression between isolines of pairs were found but found not significant in differential expression) (Table 1). Twelve DEGs were found common between NIL_ 3 and NIL_17 on chromosome 4 A for metribuzin resistance (Fig. 1). Common DEGs in the two NIL pairs from the targeted 4 A QTL were used for metribuzin resistance analysis. Genes *TraesCS4A03G0812600*, *TraesCS4A03G0819500*, and *TraesCS4A03G0912300LC* were consistently downregulated (S-isolines had higher expression than R-isolines) in the metribuzin treatment but not detected as DEGs in the control. Three significantly different upregulated genes (R-isolines had higher expression than S-isolines) (P≤0.05) were located just outside the 4 A QTL (Table S1): *TraesCS4A03G0542300*, *TraesCS4A03G0558000*, and *TraesCS4A03G0741300* (Table 2). Similarly, *TraesCS4A03G1099000*, *TraesCS4A03G1171000*, and *TraesCS4A03G1181300* were upregulated (R-isolines had higher expression than S-isolines) and significantly differed between isolines in the metribuzin treatment within the 4 A QTL. These nine DEGs identified within and near the 4AQTL in NIL pairs can be considered as candidate genes for metribuzin resistance. The details of their molecular functions are in Table 2 and Table S1. However, the control and treatment groups had no common DEGs within the targeted QTL, as treatment DEGs did not significantly differ (P > 0.05) from control DEGs.

**Table 1:** Gene expression obtained from RNA sequencing between R-isolines and S-isolines of two pairs of near-isogenic lines (NILs) across the whole genome, on chromosome 4 A and within *Qsns.uwa.4 A.2*

Treatment	Expression pattern	NIL pairs								
		NIL_3			NIL_17			Common		
		Genome	Chromosome 4A		Genome	Chromosome 4A		Genome	DEGs on Chromosome 4A	DEGs within QTL*
Control	Upregulated	5164 (3.7%)	282 (4.2%)		1276 (0.9%)	51 (0.7%)		113 (0.1%)	2 (0.03%)	0
	Downregulated	4698 (3.3%)	244 (3.6%)		1184 (0.8%)	69 (1.0%)		86 (0.1%)	5 (0.1%)	3
	Total	9862 (7.0%)	526 (7.9%)		2460 (1.7%)	120 (1.8%)		199 (0.1%)	7 (0.1%)	3
Treatment	Upregulated	1852 (1.3%)	138 (2.0%)		2371 (1.7%)	110 (1.6%)		59 (0.04%)	6 (0.1%)	3
	Downregulated	3456 (2.4%)	203 (3.0%)		3931 (2.8%)	171 (2.5%)		238 (0.2%)	6 (0.1%)	3
	Total	5308 (3.7%)	341 (5.1%)		6302 (4.5%)	281 (4.1%)		297 (0.2%)	12 (0.2%)	6

* QTL *Qsns.uwa.4 A.2* is within the interval markers Xbarc170 and Xgwm350. ~138,790 genes detected according to the reference genome. The number of genes is calculated by dividing ~ 138,790/21 = ~ 6609 to calculate the percentage of upregulated and downregulated genes on chromosome 4 A. The values in brackets are the proportion of genes in the whole genome or on chromosome 4 A, calculated from the number of DEGs divided by the total number of genes detected in the transcriptomic analysis in the whole genome or on chromosome 4 A

**Table 2:** Differentially expressed genes in isolines common to the two near-isogenic line (NIL) pairs on chromosome 4 A and their annotated functions based on RNA sequencing

Genes	log_2_FC NIL_3	log_2_FC NIL_17	Gene start (bp)	Gene end (bp)	Gene function/annotation
***TraesCS4A03G0812600*** **(** ***TraesCS4A02G326400*** **)**	–2.96	–3.49	612,401,995	612,403,260	Ethylene-responsive transcription factor
***TraesCS4A03G0819500*** **(** ***TraesCS4A02G330100*** **)**	–7.01	–3.09	613,948,551	613,949,732	Leucine-rich repeat (LRR)-serine/threonine protein kinase
***TraesCS4A03G0912300LC*** (***TraesCS4A02G540400LC***)	–5.06	–2.51	641,848,507	641,849,641	Uncharacterised
***TraesCS4A03G1099000*** (***TraesCS4A02G440700***)	2.11	1.54	714,457,633	714,463,236	Nitrate excretion transporter (NAXT)
***TraesCS4A03G1171000*** (***TraesCS4A02G466000***)	3.78	2.16	737,488,947	737,493,663	Disease resistance protein RGAs/NB-LRR-receptor kinase
***TraesCS4A03G1181300*** (***TraesCS4A02G469600***)	8.53	2.74	739,710,098	739,711,681	Aspartyl protease
*TraesCS4A03G0741300* (*TraesCS4A02G290900*)	1.27	1.23	593,189,940	593,190,850	Glycine-rich protein; responss under osmotic stress
*TraesCS4A03G0558000* (*TraesCS4A02G210100*)	1.07	1.07	503,197,597	503,199,959	Flaggerin, di-valent metal binding protein
TraesCS4A03G0542300 (*TraesCS4A02G203000*)	1.46	1.4	492,653,225	492,654,122	Dirigent protein 1-like; related to stress resistance
*TraesCS4A03G0474900* (*TraesCS4A02G173800*)	–1.11	–2.61	440,895,030	440,896,312	Xylanase inhibitor protein 1
*TraesCS4A03G0276500* (*TraesCS4A02G130300*)	–1.28	–1.43	170,881,259	170,897,924	Phospholipid-transporting ATPase 1
*TraesCS4A03G0141900* (*TraesCS4A02G070600*)	–1.52	–2.62	67,978,564	67,980,472	UDP-glycosyltransferase

Note: Genes in bold are in the *Qsns.uwa.4 A.2* marker interval between Xbarc170 and Xgwm350, with a physical position of 607,888,155–739,865,914 bp. The screening marker Xbarc343 (physical position 694,903,704 bp) is within the QTL interval. Upregulated genes (positive value) are those expressed more in resistant (R) isolines, and downregulated genes (negative value) are those expressed more in susceptible (S) isolines. Log_2_FC is the Log_2_fold change of the DEGs. Genes in brackets () are from RefSeq1.1.

In the control, three DEGs—*TraesCS4A03G0781400* (*TraesCS4A02G312800*), *TraesCS4A03G0990600* (*TraesCS4A02G398300*), and *TraesCS4A03G1005600* (*TraesCS4A02G404400*)—were detected in both NIL pairs and significantly downregulated in the control, but not detected in either of the NIL pairs after the metribuzin treatment. This could be due to higher gene expression in R-isolines in response to treatment, resulting in non-DEGs (differ in gene expression but not-significant, P > 0.05) between the R-isolines and S-isolines. This outcome strongly suggests that differences in genetic backgrounds between isolines may cause different gene expressions in response to metribuzin treatment.

### Single nucleotide polymorphisms (SNPs) between R and S isolines

We detected 4,051 SNPs in the R-isolines and S-isolines from all chromosomes. Five SNPs occurred within the 4 A QTL (Table 3; Tables S2 and S3) that mostly differed between the R-isolines and S-isolines, with three detected within genes (non-DEGs) and two found close to the candidate genes.

**Table 3:** Single nucleotide polymorphism (SNP) detected in isolines of NIL pairs within the *Qsns.uwa.4 A.2* region

Variant type	Reference seq. variant	Variant (R or S)	Variant physical position (bp)	Associated gene ID	Annotation	Variant position	Overlapping lines
SNP	G	T	709,770,189	*TraesCS4A03G1080800*	Cellular homeostasis	Within gene (not a DEG)	3S_C and 17S_C
SNP	A	G	**714,355,066**	*TraesCS4A03G1099000* (DEG)	Nitrate excretion transporter (NAXT)	0.10 Mbp from DEG	3S_C, 3S_T, and 17S_C
SNP	T	C	724,909,357	*TraesCS4A03G1133400*	Biosynthesis of secondary metabolites	Within gene (not a DEG)	3R_C and 17R_C
SNP	A	G	726,024,541	*TraesCS4A03G1138500*	Glutathione S-transferase	Within gene (not a DEG)	3R_T and 3S_T
SNP	C	T	739,694,493	*TraesCS4A03G1181300* (DEG)	Aspartyl protease	0.015 Mbp from DEG	3R_T, 3R_C, and 17R_C

Note: Differentially expressed genes (DEGs) with SNPs common to both NIL pairs are in bold. bp is base pairs. 3R and 17R are resistant isolines, 3 and 17 S are susceptible isolines, C is control, and T is treatment. An SNP at a position marked with bold can be used as a marker for metribuzin resistance breeding

A major SNP common between two NIL pairs occurred near (0.10 Mb away) the candidate gene *TraesCS4A03G1099000*. Another SNP was found 0.015 Mb from candidate gene *TraesCS4A03G1181300* (Table 3; Tables S2 and S3) but was not polymorphic between all R-isolines and S-isolines and thus cannot be considered a true SNP (Table 3). No other variants from 4 A chromosome common to NIL pairs were detected (Table 3; Tables S1–S3).

### Gene ontology and functional annotation of DEGs

**Fig. 2:**
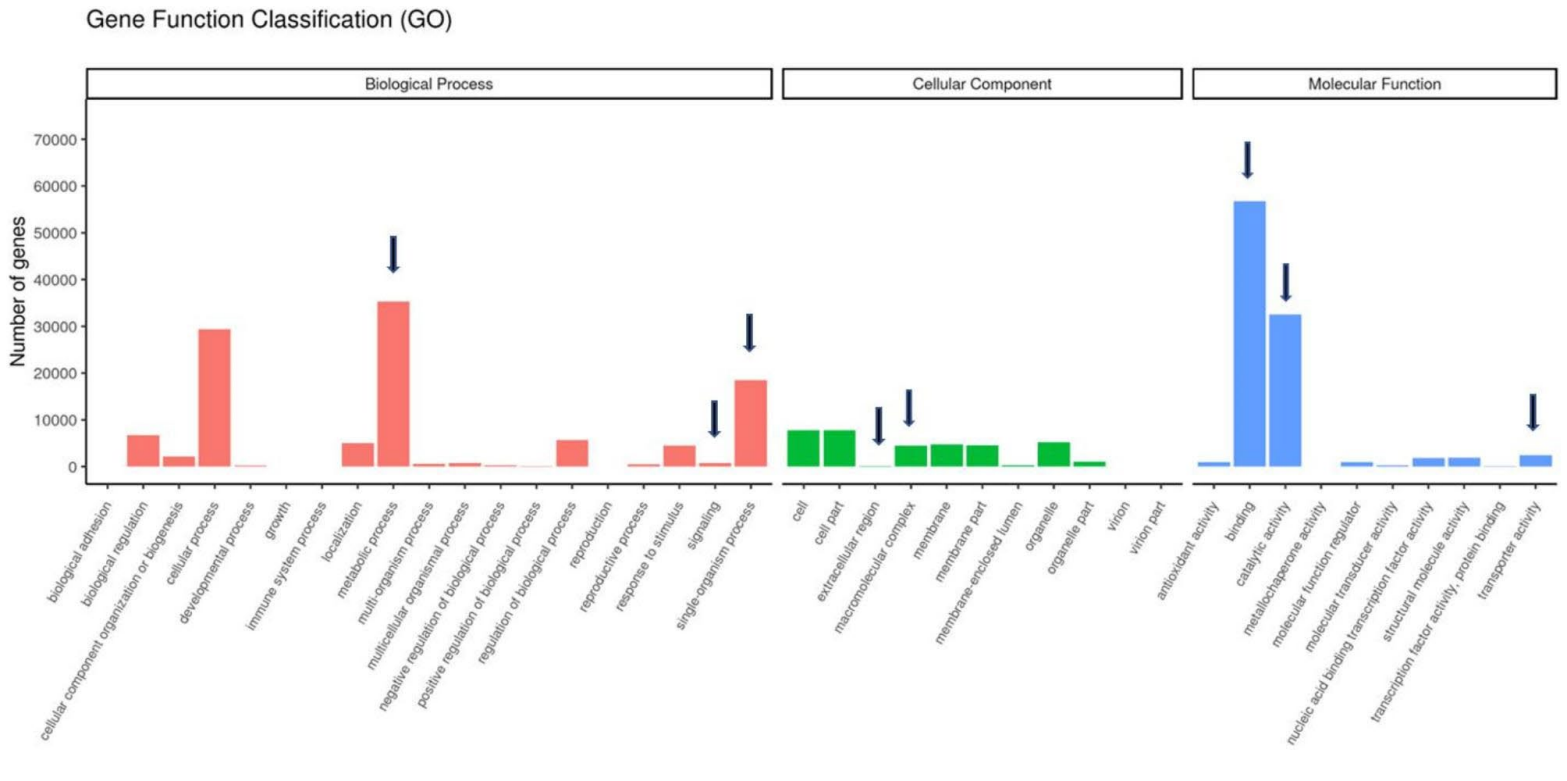
Gene function classification (GO) based on biological function, cellular components, and molecular function. The most relevant biological processes in common DEGs are responses to stimulus, localisation, and signaling. The most relevant to cellular components are extracellular region and macromolecular complexes, while those most relevant to molecular functions are specific site binding and catalytic and transporter activities (indicated with arrows)

Biological processes, cellular components, and molecular functions were used to describe GO functions. The highly expressed genes in the metribuzin treatment are presented with their molecular functions and biological processes (Fig. 2). The most prevalent processes were biological processes, localisation, metabolic processes, response to stimuli, signaling and cellular processes. The most common cellular components were extracellular regions, macromolecular complex, membranes, and organelle parts. The most common molecular functions—binding, catalytic and transporter activities (Fig. 2 and Table S4)—were found to be significant in the metribuzin treatment.

**Fig. 3:**
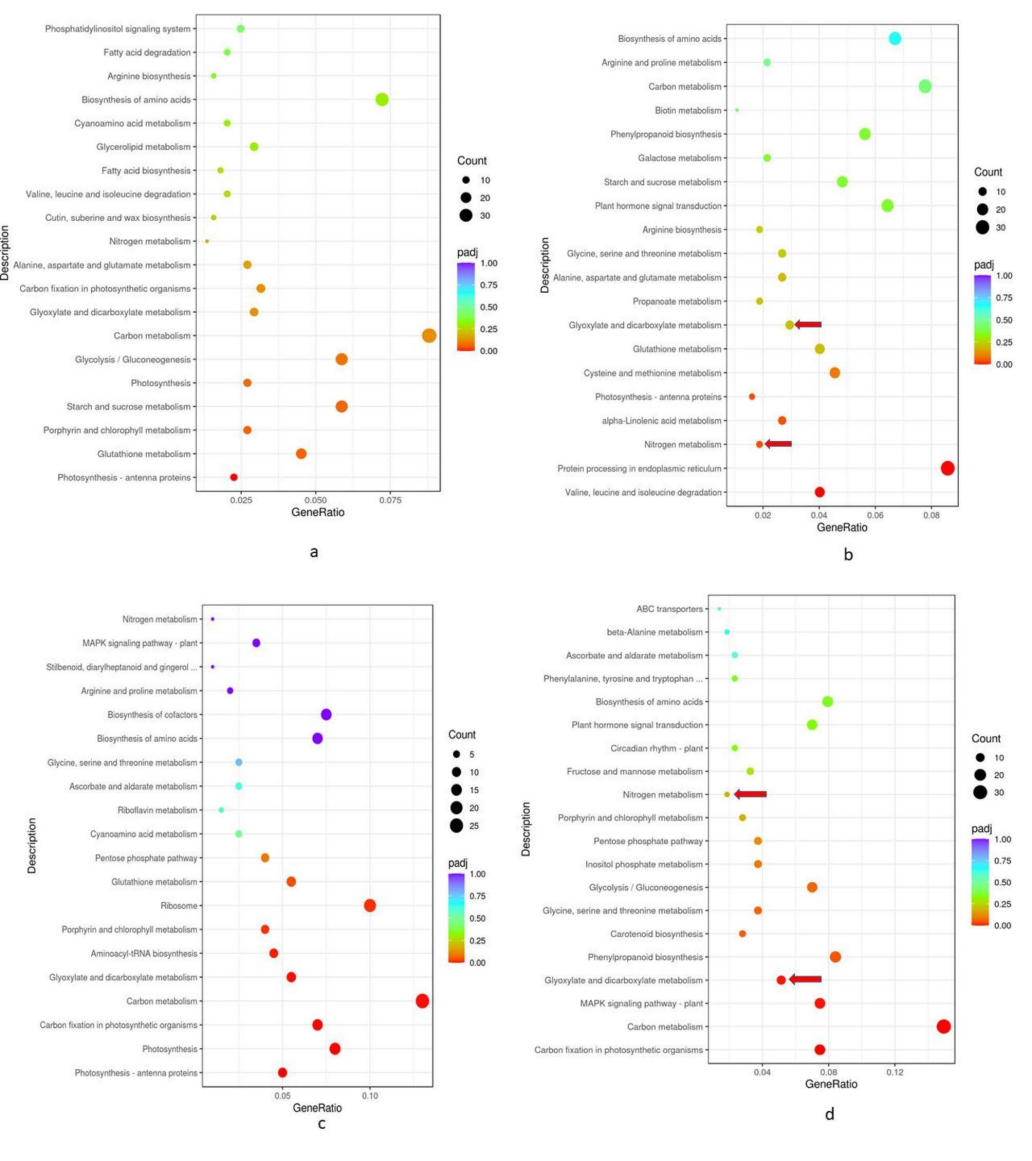
Kyoto Encyclopedia of Genes and Genomes (KEGG) pathway enrichment analysis of differentially expressed genes (DEGs) in near-isogenic lines in the control and metribuzin treatments. (a) NIL_3 in the control; (b) NIL_3 in the metribuzin treatment; (c) NIL_17 in the control; (d) NIL_17 in the metribuzin treatment. Padj. is the corrected probability (P) value; red dots indicate highly significant enriched pathways (Padj.≤0.01), light yellow dots indicate significantally enriched pathways (Padj.≤0.05). Arrows indicate the pathways common to NIL_3 (b) and NIL_17 (d) pairs; KEGG analysis significantly enriched nitrogen metabolism and glyoxylate-decarboxylate metabolism in the metribuzin treatment [67]

For the metribuzin treatment, KEGG pathway enrichment analysis of DEGs in isolines identified common pathways for glyoxylate and decarboxylate metabolism(KEGG ID; bdi00630), and nitrogen metabolism(KEGG ID; bdi01200) (Fig. 3). The phenylpropanoid and mitogen-activated protein kinase (MAPK) pathways(KEGG ID; bdi00940 and bdi04016, respectively) were the most significantly stimulated pathways for NIL_3. In contrast, alpha-linolenic acid and nitrogen metabolism were the most stimulated pathways for NIL_17 (Fig. 3).

### Candidate gene validation using RT-qPCR

**Fig. 4:**
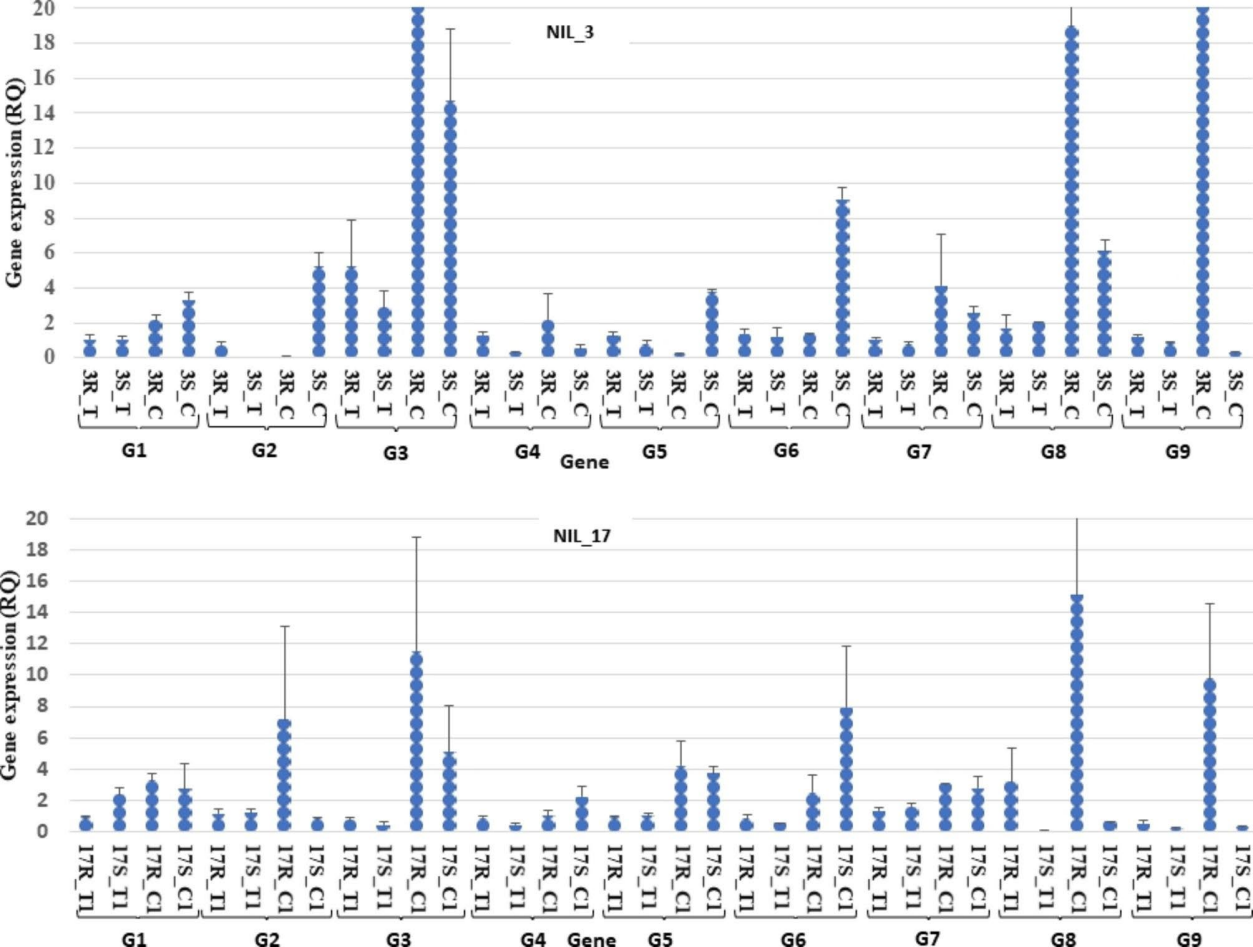
Gene expression analysis using RT-qPCR in NIL_3 and NIL_17 in the form of relative quantification (RQ) using dotplots (blue dots). 3R_T and 3S_T are the RQ of gene expression in resistant and susceptible isolines of NIL_3 (treatment), 17R_T and 17S_T are the RQ of gene expression in resistance and susceptible isolines of NIL_17 (treatment), 3R_C and 3S_C are the RQ of gene expression in resistance and susceptible isolines of NIL_3 (control), and 17R_C and 17S_C are the RQ of gene expression in resistance and susceptible isolines of NIL_17 (control). G1, G2, G3, G4, G5, G6, G7, G8, and G9 are the genes used for gene validation using RT-qPCR: *TraesCS4A03G0812600* (G1), *TraesCS4A03G0819500* (G2), *TraesCS4A03G0912300LC*(G3), *TraesCS4A03G1099000 (G4)*, *TraesCS4A03G1171000* (G5), *TraesCS4A03G1181300* (G6), *TraesCS4A03G0542300* (G7), *TraesCS4A03G0558000* (G8), and *TraesCS4A03G0741300* (G9)

Nine candidate genes that significantly differed between isolines in the RNA sequencing-based DEG analysis from 4 A QTL (Table 2) were used in RT-qPCR-based DEG analysis (Fig. 4; Table S1 and Table S5). In the RT-qPCR analysis, DEGs were categorised based on the ratio of relative quantification (RQ) in gene expression between R-isoline and S-isoline significance at P≤0.05 (Table S5) using the paired t-test. Among the nine candidate genes, *TraesCS4A03G1099000* (nitrate excretion transporter) was upregulated significantly, whereas *TraesCS4A03G1181300* (aspartyl protease) and *TraesCS4A03G0741300* (glycine-rich proteins) were upregulated (but non-significant) in both NIL pairs.

### Gene network analysis using candidate genes

**Fig. 5:**
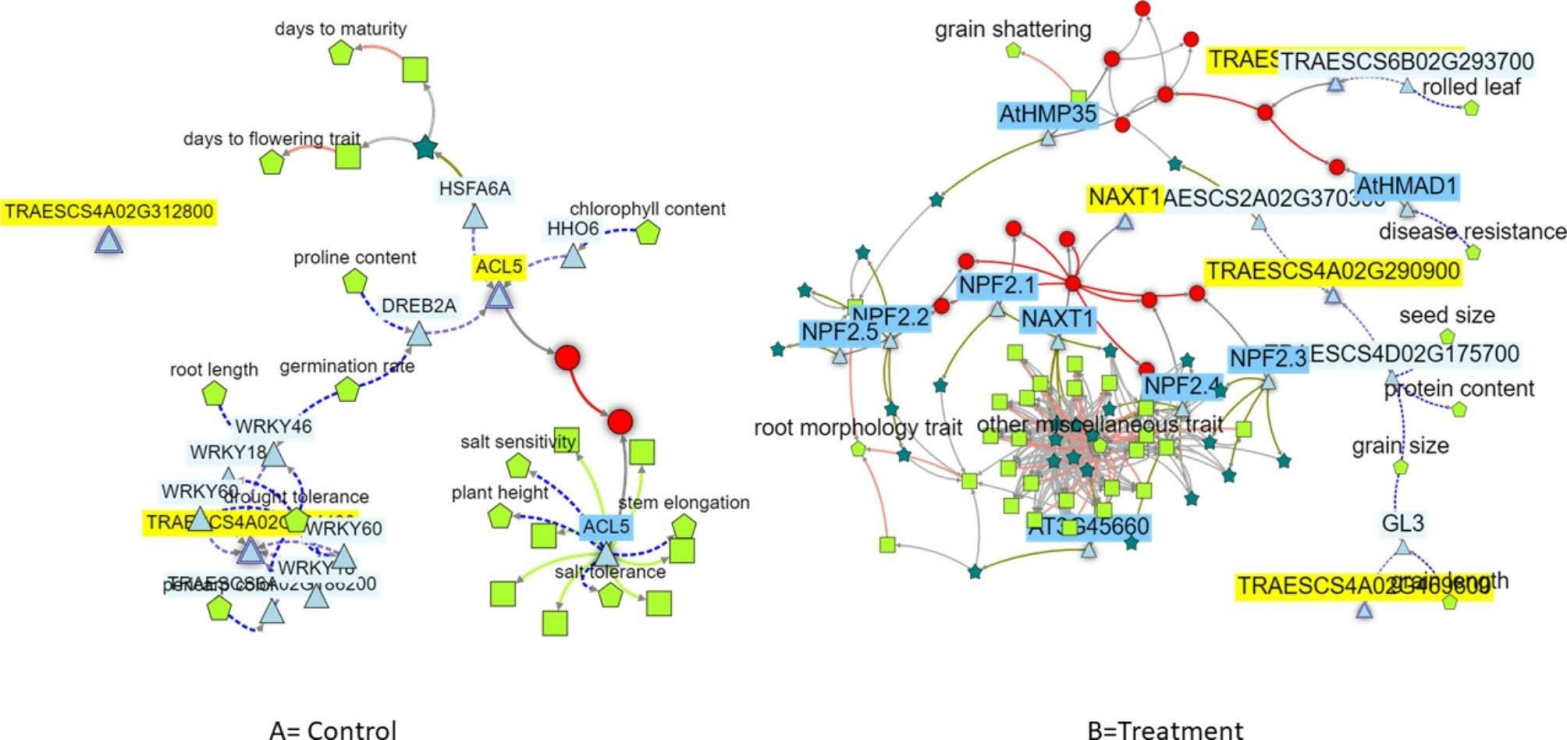
Gene network analysis using DEGs obtained from the control (A) experiment: *TraesCS4A02G312800*, *TraesCS4A02G398300*, and *TraesCS4A02G404400*, and DEGs obtained from the treatment (B) experiment: *TraesCS4A02G440700*, *TraesCS4A02G469600*, *TraesCS4A02G210100*, and *TraesCS4A02290900*. Triangle, genes directly associated with metribuzin resistance; hexagon, gene isoforms; square, phenotypes may relate to metribuzin resistance; red circle, proteins; dark green star, interaction network of resistance genes; red lines, protein–protein interactions that may interact with metribuzin resistance; light red lines, interaction of phenotypes may relate to metribuzin resistance

In the control, the major DEGs—*TraesCS4A03G0781400*, *TraesCS4A03G0990600*, and *TraesCS4A03G1005600* (named *TraesCS4A02G312800*, *TraesCS4A02G398300*, and *TraesCS4A02G404400* in RefSeq1.1, respectively)—were used for network analysis. In the metribuzin treatment, the major DEGs—*TraesCS4A03G1099000, TraesCS4A03G1181300*, *TraesCS4A03G0558000*, and *TraesCS4A03G0741300* (named *TraesCS4A02G440700*, *TraesCS4A02G469600*, *TraesCS4A02G210100*, and *TraesCS4A02290900* in RefSeq1.1, respectively)—showed consistent expression patterns in the RNA-seq and RT-qPCR experiments and were used for network analysis. The gene network analysis revealed the interaction of these genes for stress resistance, including disease resistance, and their contribution to traits such as seed size, root morphological traits, and protein content (Fig. 5).

## Discussion

### Differentially expressed candidate genes underlying the *Qsns.uwa.4 A.2* region

Using two NIL pairs with different genetic backgrounds in the DEG analysis using RNA-seq, we identified nine candidate genes and SNPs from *Qsns.uwa.4 A.2* which identified six upregulated (higher gene expression in R-isolines than S-isolines) and three downregulated (higher expression in S-isolines than R-isolines) candidate genes near and within the *Qsns.uwa.4 A.2*, which were validated by RT-qPCR. The candidate genes are related to carbon metabolism, lipid metabolism, protein metabolism, and stress resistance. No common DEGs between the treatment and control were detected on chromosome 4 A (Table S1), suggesting that the applied metribuzin affected isolines gene expression. The DEGs found common between two NIL pairs from all wheat chromosomes are listed in Table S6.

Recent studies using Synthetic W7984 × Opata M85 QTL mapping [8], transcriptomic analyses of Chuan Mai 25 and Ritchie parents [16], and QTL mapping by DArT sequencing and genotyping in Chuan Mai 25 × Ritchie population [7] reported candidate genes for metribuzin resistance in wheat, mainly associated with photosynthesis, photosystem, and transporters related to the biosynthesis of fructose, mannose, galactose, and nucleotides. Similar functional categories genes are discussed in the following paragraphs.

Candidate gene *TraesCS4A03G0812600* is an ethylene-responsive factor (ERF) with domain (AP2/ERF). The ERF domain has diverse roles in biotic and abiotic stresses [17]. *TraesCS4A03G0812600* has the conserved domain amino acid sequences (rgvrkrpwg) at the 14th position, possibly the gene is ERF-1. ERF-1 expression depends on S-adenosyl- methionine (SAM) whose regulation is usually determined by the metabolism of methionine [18]. The major roles of SAM are regulating DNA methylation and 1-aminocyclopropane-1-carboxylic acid (ACC) synthase. In the absence of the ACC molecule, inactivated ETR-1 regulates the degradation of the C-terminal end of EIN-2 (a protein kinase) resulting in *constitutive triple response 1* (*CTR1*) expression, helping in stopping ethylene production [19, 20]; whereas higher expression of *ctr1* gene prevents EIN-3 degradation and promotes further ethylene signaling and biosynthesis, and thus may enhance leaf senescence. Dihydroxymethylchlorophyllide is a methyl derivative, is synthesised from ethylene, actively involves in chlorophyll biosynthesis, plays a significant role in chlorophyll subunit (CHLH) of Mg-chelatases [21] and seperation, and eventually degrading the green pigments from leaves. However, gene expression result was found inconclusive from RT-qPCR for this gene as there was no significant differences between isolines (Table S5 and Fig. 4). From RNA-seq, gene expression was found significantly different in isolines and found common to the isolines of both NIL pairs, we speculated that this gene may express significantly different between isolines when severe and longer metribuzin stress is imposed, when degradation of chlorophyll is started.

The gene *TraesCS4A03G0819500* had higher expression in S-isolines than R-isolines with molecular functions similar to leucine-rich repeat (LRR)-serine/threonine-protein kinase. This gene may recognise and transduce metribuzin signals toward the effectors. LRR regulates various abiotic and biotic stresses in plants [22–24], with a major role in transducing metribuzin signals, through the membrane, triggering the MAPK cascade for synthesising jasmonic acid, flavonoids, and phenylpropanoids [25, 26] and determining ethylene signaling. Ethylene response factor, a downregulated candidate gene (Table 2), also regulates the constitutive triple response-1 (ctr1), which has a functional domain similar to serine/threonine protein kinase [26, 27], and may closely resemble kinase activities, such as phosphorylation in serine and threonine (ST) residues [27, 28]. This process of phosphorylation leads to conformational changes [29, 30]. A gene ST8 on PSII-component phosphorylation/activation-deactivation has been reported [31]. When the metribuzin signal passes through phosphorylation, mutation of the *Psb* gene and substitution of amino acids may occur at the Ser-264-Thr site [32], which codes the D1 protein, affecting the efficiency of electron transport and ATP synthesis. ATPs are important for cell differentiation, photosynthetic processes, cell growth, and various cellular metabolism.

Candidate gene *TraesCS4A03G1171000* is a resistance gene analog-5 (RGA-5), expressed more in R-isolines than S-isolines. Plant resistance gene analogs (RGAs) also have diverse roles in determining biotic and abiotic resistance in plants [33]. Well-known RGAs are nucleotide-binding site leucine-rich repeats (NB-LRR), receptor-like kinases, and receptor-like proteins. These protein-encoding genes are responsible for receiving signals through receptors and transducing signals to nucleotide-binding site(s) for transcription [34] [35]. Similarly, the upregulated NB-LRR gene also produces ROS, involved in lignin and callus biosynthesis [36]. The lignification-related gene *TraesCS4A03G0558000* was also upregulated in both NIL pairs but detected outside the QTL (Table S1). These complex biopolymers (lignin and calluses) may be used as energy suppliers through the tricarboxylic acid cycle (TCA) when plants are under stress and may also help with the turnover of PS-II components to their original state. Succinyl CoA, an intermediate of TCA cycle with amino acid glycine initiate the synthesis of chlorophyll-A [37] during chlorophyll biosynthesis, therefore, *TraesCS4A03G0558000* may also help in chlorophyll biosynthesis.

An annotation of the common (between two NIL pairs) downregulated *TraesCS4A03G0912300LC* was unavailable. The sequence similarity analysis, GO, UniProt search, NCBI blast, and knetminer analyses did not reveal any links to other genes. The candidate gene is present within 4AQTL with other candidate genes, indicating that they may follow a similar pathway of metribuzin resistance [38]. Therefore, we assume the gene is related to metribuzin resistance.

Candidate gene *TraesCS4A03G1099000* showed upregulation with gene function related to the nitrate excretion transporter. The RT-qPCR and RNA-seq based DEG analysis revealed that this gene significantly differed between R-isolines and S-isolines in NIL_3 (Table S5, Fig. 4, and Table 2). None of the other candidate genes significantly differed in their expression between isolines; therefore, this gene may be the first responsive gene to metribuzin (e.g., signal-perceiving or signal-activating). The functional role of this gene in plants is directly associated with stress response, signaling, growth, and metabolism of peptides and proteins [39]. Carbohydrate and protein metabolisms are also related to determining the nitrate level in plants. Photosynthate portioning, carbon–nitrogen balance, maintaining cell homeostasis, signal transduction, and nucleotide synthesis and repairs are crucial processes under stress conditions [40, 41] and related to nitrogen metabolism. Similarly, functional roles of anions with the oxygen-evolving complex, a PS-II component, have been reported; the efficiency of anions with CaMn_4_O_5_ of PS-II were ordered Cl > Br > NO_3_ > NO_2_ > I [42–44]. It has been reported that NO_2_ is oxidised by the OEC of PS-II, thus inhibits the PS-II [45]. Hebicides including of photosynthestic inhibitors also interfere in the normal nitrate reduction process which can result in nitrate accumulation in PS-II [46], which may also hamper the ammonia assimilation in the cells. Important gene/protein such as Qb site from PS-II, a reported site for herbicide binding [47], could be the same site where nitrate may interact for metribuzin resistance. The overly produced nitrates in plant cells under metribuzin stress may change the expression of gene *TraesCS4A03G1099000* (nitrate excretion transporter), may help to release nitrate from PS-II for ammonia assimilation process [48, 49] and signaling other linked genes such as nitrite reductases (NR), glutamine synthetase (GS), glutamate synthase (GOGAT) [50], and chlorophyll biosynthesis [51–55]. An SNP (marked bold in Table 3) identified near the candidate gene *TraesCS4A03G1099000* could be used to select for metribuzin resistance in wheat. While this SNP was not polymorphic between R-isolines and S-isolines of NIL_17 under metribuzin treatment (Table S2)—possibly because the metribuzin effects at this SNP site result in the substitution of DNA sequences from G to A in isoline 17S_T—this change may contribute to metribuzin resistance in isoline 17S_T. This mutation could be one of the reasons explaining why *TraesCS4A03G1099000* expression did not significantly differ in contrasting isolines of pair NIL_17 in the RT-qPCR. However, this SNP was polymorphic between R-isolines and S-isolines in NIL_3; mutation (A to G) at this SNP site in NIL_3 (Table S3 and Table 3) may favour to bind with metribuzin in 3S_T isoline, with significant differences in gene expression (*TraesCS4A03G1099000*) observed between isolines in NIL_3. Gene *TraesCS4A03G1099000* was also closely associated (Table 3) with marker, Xbarc343 (physical position 694,903,704 bp on 4 A chromosome), which was used in NIL development [15] for metribuzin resistance, and SNP site was detected (in this current research) at 714,355,066 bp; therefore, the marker distance of 19.45 Mb has decreased from 694,903,704 bp (original Xbarc343 position) to 714,355,066 bp (current SNP position). This SNP position at 714,355,066 bp is 0.10 Mbp away from the candidate gene *TraesCS4A03G1099000*, may affect the metribuzin resistance. Using this SNP as a molecular marker, other key linked variants can be identified using higher resolution mapping for selecting metribuzin resistance.

Upregulated candidate gene *TraesCS4A03G1181300* was common to both NIL pairs, with the most significant differences in gene upregulation from RNA-seq (Table 2) and upregulated through RT-qPCR analysis (Table S5). It has a function related to aspartyl protease [56] found in chloroplast membranes and stroma [57], and its processing is senescence-related [58]. Aspartate, a amino acid closely resembles to glutamate, are frequently transaminated [59] during amino acid synthesis, while glutamate is a precurser for chlorophyll synthesis [60].

Another candidate gene *TraesCS4A03G0741300* was responsive to stress; the functional annotation revealed that the gene encodes glycine-rich protein, related to stress resistance and protein processing [60, 61]. During chlorophyll biosynthesis succinyl CoA, an intermediate of Krebs cycle and the amino acid glycine initiate the synthesis of chlorophyll-A leading to production of protochlorophyllide or protochlorophyll [62–64] which are the initiating molecules in chlorophyll biosynthesis. This gene was significantly upregulated in the RNA-seq and upregulated in RT-qPCR in both NIL pairs (G9 gene in Fig. 4), suggesting an important role in chlorophyll synthesis and metribuzin resistance in wheat.

Another upregulated DEG common between two NIL pairs is *TraesCS4A03G0558000* with annotation function is related to divalent metal-binding) (Table S1). Gene *TraesCS4A03G0558000* could be the Mg^++^ion of chlorophyll porphyrin ring, because it likely follow the same pathway of chlorophyll synthesis and metribuzin resistance as with other candidate genes from the selected QTL. Mg^++^ binding protein (gene *TraesCS4A03G0558000*) makes bonds with methyl groups and four nitrogen atoms in square planner arrangement in chlorophyll molecule [65]. Gene higher expression may directly be associated with the formation of chemical energy through the process of [66]. However, gene was not found commonly expressed to both pairs in RT-qPCR (G8 gene in Fig. 4), due to differential level of chlorophyll may present in NIL pairs leaves after 15 days of metribuzin treatment.

Gene network analysis using DEGs from the control and metribuzin treatments revealed that candidate genes were responsive to stresses such as disease and some agronomically important traits such as grain size, root traits, and protein content (Fig. 5). Our previous study [15] found that the 4 A QTL responsible for metribuzin resistance was closely linked to thousand-grain weight. Similarly, the network analysis in the present study also found that the key candidate genes underlying 4AQTL may play roles in regulating grain size, suggesting that targeting this QTL may improve metribuzin resistance and the yield component trait simultaneously.

Candidate genes responsive to metribuzin treatment, genes such as *TraesCS4A03G1099000* (nitrate excretion transporter), may act as a precurser for chlorophyll synthesis genes, may help to activate the genes *TraesCS4A03G1181300* (aspartyl protease), *TraesCS4A03G0558000* (divalent metal binding protein), *TraesCS4A03G0741300* (glycine-rich protein) and *TraesCS4A03G0812600* (ethylene-responsive). During metribuzin stresss, gene *TraesCS4A03G1099000* may get express and release from PS-II in response to stress, and may activate the other precursers such as aspartate, glycine, metal binding protein, and ethylene responsive genes for chlorophyll synthesis which eventually may help in photosynthetic carbon assimilation and metribuzin resistance which is also revealed from the thousand grain weight (TGW) and metribuzin resistance were found closely linked genes in [15]. Therefore, it can be said while selecting for the gene *TraesCS4A03G1099000*, using linked molecular marker, we can able to balance the carbon-nitrogen content in wheat, which ultimately helps in carbon assimilation, chlorophyll enhancement and metribuzin resistance in cyclic manner. Other candidate genes such as *TraesCS4A03G0819500* (leucine-rich repeat (LRR)-serine/threonine-protein kinase) and *TraesCS4A03G1171000* (nucleotide-binding (NB)-LRR receptor kinase), may also be involved in pathways of metribuzin resistance when longer duration of metribuzin dose is applied.

The KEGG analysis [67–69] reveaed that the candidate genes were involved in nitrogen metabolism and carbohydrate metabolism (Fig. 3) which were accordance with the results (candidate genes function; Table 2) obtained in DEG analysis, indicating that candidate genes are involved in carbon-nitrogen related pathways for metribuzin resistance. Genes that significantly differed in their expression in NILs in the RNA-seq but not validated in the RT-qPCR analysis may still be involved in metribuzin resistance pathways, requiring more metribuzin exposure time to express differentially between isolines. Therefore, further a time-point experiment using RT-qPCR is needed to validate other candidate genes for metribuzin resistance in wheat. This study reported and validated DEGs using RNA-seq and qPCR, which indicated that *TraesCS4A03G1099000* (nitrate excretion transporter), *TraesCS4A03G1181300* (aspartyl protease), and *TraesCS4A03G0741300* (glycine-rich proteins) are the major genes for metribuzin resistance in wheat.

### Variants in the two NIL pairs

Five variants within the 4 A QTL differed between the isolines for both NIL pairs, including three SNPs within the genes (non-DEGs) and two SNPs away from candidate genes. SNPs common between isolines of NIL pairs are the most valuable markers as they also indicate similar genomic composition between two different genetic backgrounds from the targeted genomic regions.

A major SNP located near gene *TraesCS4A03G1099000* can be considered a marker, which was within gene *TraesCS4A03G1098900* (Table 3). Both these genes code for the nitrate excretion transporter. This SNP overlapped in isolines of NIL pairs (Table S3), which could affect the expression of candidate gene *TraesCS4A03G1099000* and may also be linked with other candidate genes. The SNP near the candidate gene (< 1Mbp) or candidate gene regulatory regions could be valuable markers for selecting metribuzin resistance. The alleles can be used for fine mapping and gene validation experiments for metribuzin resistance in wheat.

## Conclusion

Transcriptomic analysis using NILs targeting major 4AL QTL *Qsns.uwa.4 A.2—*explaining up to 69% of the phenotypic variance for metribuzin resistance in wheat—revealed nine candidate genes and five consistent SNPs associated within the QTL. Among the candidate genes; *TraesCS4A03G1099000* (nitrate excretion transporter) and *TraesCS4A03G1181300* (aspartyl protease) and *TraesCS4A03G0741300* (glycine-rich protein) are likely major candidates for determining metribuzin resistance based on the consistent RNA-seq and RT-qPCR results. The detected SNP markers at 714,355,066 bp on wheat chromosome 4 A might be used to select important candidate gene *TraesCS4A03G1099000* for metribuzin resistance which may also ultimately help in yield enhancement in wheat.

## Methods

### Plant materials and metribuzin treatment

Two NIL pairs with different genetic backgrounds—3R and 3 S and 17R and 17 S—were used in this study (R-isolines are resistant isolines with the R allele, and S-isolines are susceptible isolines with the S allele) [15]. NILs were developed from a cross of Chuan Mai 25 and Ritchie. Chuan Mai 25 is a Chinese cultivar derived from the cross of 1414/Chuanyu 5//Genaro 80 (https://repository.cimmyt.org/xmlui/bitstream/handle/10883/1222/64613.pdf, accessed on 10 October 2022), while Ritchie is a European cultivar released in the United Kingdom. The NILs were grown in three biological replicates in the glasshouse at The University of Western Australia (UWA), Perth, Western Australia. Seeds of each isoline were sown and sprayed with 200 g ai./ha pre-emergent dose of metribuzin (recommended dose to control weeds in Western Australia wheat fields). NILs seeds were kept in the Gene Bank, UWA, Perth. The plant phenotyping/metribuzin assessment method is described in [15]. Fifteen-day-old seedlings and roots were sampled for RNA extraction. NIL pair seeds were sown under 100% field capacity in river sand (no dirt or clay). Wheat seedlings (including roots) were used for the total RNA sample collection; for example, three seedlings of NIL_3_R/S and NIL_17_R/S were bulked for Replication-I, three seedlings of NIL_3_R/S and NIL_17_R/S were bulked for Replication-II, and three seedlings of NIL_3_R/S and NIL_17_R/S were bulked for Replication-III. The samples were placed in chilled 50 mL autoclaved tubes, immediately capped, and immersed into a liquid nitrogen dewar at − 197° C to extract samples total RNAs from NIL pairs—3R_T, 3S_T, 3R_C, 3S_C, and 17R_T, 17S_T, 17R_C, and 17S_C—for gene expression analysis.

### RNA extraction, library preparation, and sequencing

Total RNA was extracted from 24 samples (four isolines × three replicates × two treatments) using an RNeasy Plus Plant Mini Kit (Qiagen) with DNase-I treatment, following the manufacturer’s instructions (Bioline, Australia). The quantity and purity of extracted RNA were assessed by NanoDrop 2000 (Thermo Fisher Scientific Inc., Australia). The cDNA synthesis, 150 bp paired-end sequencing, raw data filtering, and other sequencing parameters were set as the default, as per the Novogene protocol (en.novogene.com). Clean data were generated as FastQ files, with clean read quality checking (Q20 and Q30) and GC contents quantified. Clean sequencing data from NIL pairs were used for gene expression and DEG analysis. Paired-end sequencing data from all samples were deposited to the National Center for Biotechnology Information (NCBI) in SRA, with an accession number of PRJNA839200.

### Transcriptome assembly and mapping quality statistics

The Illumina NovaSeq6000 platform generated 183 Gb high-quality 150 bp pair-end sequencing reads from the 24 samples after quality control, with an average of 76 million clean reads per library. The percentage of bases with Phred quality scores of Q20 and Q30 were 98% and 94%, respectively (Table S7). Approximately 86% of the sequenced reads were mapped to the wheat reference genome, of which 89% per library uniquely matched. The GC base percentage was 54% of the total bases. The sequencing error rate was < 0.02, calculated using the Qphred parameter as the default, as per the Novogene protocol. The fragments per kilobase million (FPKM) mapped reads detected in each library ranged from 98,253 to 117,430 (average 111,032), accounting for 80% of wheat genes. Pearson’s correlation coefficients for each combination of samples within biological samples ranged from 0.8 to 0.9, indicating there was a minimal variation between biological replicates.

### Sequencing, analysis, and differentially expressed gene (DEG) identification

The obtained sequences were aligned to the reference sequences (RefSeq V2.1) using the HISAT2 algorithm [70, 71]. Bowtie2 was used to align the reads to the Chinese Spring wheat reference transcriptome [72], with the gene expression level calculated using RSEM (RNA-Seq by Expectation Maximisation) software v1.2.1246 with default parameter [73]. The expected number of transcripts was reported in FPKM. DEGs were detected with DEGseq as described in Wang et al. [62] with the following parameters: differential genes are Log_2_fold change > 1 for upregulated genes, and Log_2_fold change <–1 for downregulated genes at P≤0.05. Common candidate genes obtained from different NIL pairs are those in which DEGs from isolines show similar gene expression patterns (upregulation or downregulation) from both pairs. The molecular functions of DEGs were also validated with the NCBI nucleotide search by blasting the gene sequences.

### Gene ontology and functional pathways analysis

Gene ontology (GO) enrichment analysis of DEGs was implemented using the clusterProfiler v4.0 R package with bias correction [74]. Significantly enriched GO terms had corrected P ≤0.05. The Kyoto Encyclopedia of Genes and Genomes (KEGG)[67–69] was used for studying pathway enrichment to understand more functions (https://www.genome.jp/kegg).

### Variant (SNPs and indels) identification

The reference sequence from IWGSC_RefSeq_v2.1 was used to align the sample sequences for identifying variants [71] (SNPs and Indels) (Table S2 and Table S3). Variant calling was performed using GATK software v4 [75], and SnpEff software v5 was used to annotate the variant sites [76]. Specifically, after applying GATK to detect variant sites, each site was counted according to the SnpEff annotation information; for example, the variant site function was plotted statistically from three aspects: non-synonymous mutation, missense mutation, and nonsense mutation. Variants were identified by comparing R-isoline or S-isoline sequences with the reference sequences; those common to each set (three replicates) of R-isolines or S-isolines and also differed between R-isolines and S-isolines were considered true variants.

### DEGs validation using RT-qPCR analysis

Candidate genes in the targeted *Qsns.uwa.4 A.2*, commonly expressed (upregulated or downregulated) between the two NIL pairs, were selected for RT-qPCR assay. RNA concentrations of the samples were measured with NanoDrop1000 Spectrophotometer (ThermoScientific) and adjusted accordingly. Total RNA was extracted from 24 samples using a Qiagen RNeasy Plant Mini Kit according to the protocol followed by Md. Sultan Mia [77]. Total RNA (1 µg) was used to synthesise cDNA using a SensiFAST cDNA Synthesis Kit (Bioline Australia) following the manufacturer’s protocol. The RT-qPCR was performed on an ABI 7500 Fast system using a SensiFAST™ SYBR No-ROX Kit (Bioline Australia). Gene-specific primers were designed based on candidate gene exon sequences with the help of Geneious software, with the wheat *actin* gene [78] used as an endogenous control to normalise gene expression. Primers were designed from the concatenated sequence regions covering the exon–exon junctions to avoid residual gDNA amplification. The function of the designed primers was checked with NCBI nucleotide blast to ensure the selected primers represent the targeted genes (Table S5). Three biological replicates were used in each isoline from two NIL pairs for treatment and control conditions. Amplification was conducted in a 20 µL reaction mix containing 10 µL of 2×SensiFAST SYBR Lo-ROX mix, 0.8 µL of 10 µM each for forward and reverse primers, 100 ng cDNA (usually 1 µL), with the following cycling protocol: 1 cycle of 95° C for 2 min, 40 cycles of 95° C for 5 s, and 63° C for 30 s. Ct mean values were calculated based on differences between the endogenous control and each sample value (ΔΔCт) converted to Log_2_ values, representing the relative quantification (RQ) for gene expression [79]. The observed values of relative quantification were analysed using paired two sample t-test. Differences in gene relative quantification between R-isolines and S-isolines determines gene upregulation (positive values)and downregulation (negative values). The parameter was set at P ≤ 0.05 for the paired t-test.

## Electronic supplementary material

Below is the link to the electronic supplementary material.

Supplementary Material 1

Supplementary Material 2

Supplementary Material 3

Supplementary Material 4

Supplementary Material 5

Supplementary Material 6

Supplementary Material 7

## Data Availability

The datasets generated and/or analysed (RNA-seq data) during the study are available in the NCBI repository with Bioproject number PRJNA839200 (released on 9 October 2022). Other data such as SNP information are available in the supplementary tables.

## Abbreviations

AP2/ERF: AP2-like ethylene-responsive
4AL: Long arm of chromosome 4 A
ATP: Adenosine triphosphate
DNase I: Deoxyribonuclease I
cDNA: Complementary DNA
DEG: Differentially expressed gene
ETR: Ethylene response
EIN: Ethylene insensitive
FPKM: Fragments per kilobase of exon per million mapped reads
G × E: Gene by environment interaction
GO: Gene ontology
HIF: Heterogeneous inbreed family
Indel: Insertion and deletion
IWGSC: International Wheat Genome Sequence Consortium
Kbp: Kilobase pair
KEGG: Kyoto Encyclopedia of Genes and Genomes
Mbp: Megabase pair
MQTL: Meta-QTL
NBS-LRR: Nucleotide-binding site leucine-rich repeat
NCBI: National Center for Biotechnology Information
NIL: Near-isogenic line
Q30: Quality score of 30
RT-qPCR: Real-time quantitative polymerase chain reaction
QTL: Quantitative trait locus
RGA: Resistance gene analog
RNA-seq: RNA sequencing
ROS: Reactive oxygen species
S: Susceptible
SNT: Serine/threonine protein kinase
SNP: Single nucleotide polymorphism
SRA: Sequence read archive
R: Resistant
SAM: S-adenosyl- methionine
TFs: Transcription factors
UTR: Untranslated region
ΔΔCT: Delta delta C (T) method
